# Comparing ultrasound-guided modified thoracoabdominal nerves block through perichondrial approach with oblique subcostal transversus abdominis plane block for patients undergoing laparoscopic cholecystectomy: a randomized, controlled trial

**DOI:** 10.1186/s12871-023-02106-z

**Published:** 2023-04-27

**Authors:** Ayşegül Bilge, Betül Başaran, Başak Altıparmak, Tayfun Et, Muhammet Korkusuz, Rafet Yarımoğlu

**Affiliations:** 1 Department of Anesthesiology and Reanimation, Faculty of Medicine, Karamanoğlu Mehmetbey University, Üniversite Mh. Şehit Ömer Halis Demir Caddesi Blok No:7, Karaman, Turkey; 2 Department of Anesthesiology and Reanimation, Muğla Sıtkı Koçman University, Menteşe Muğla, Turkey; 3 Department of Anesthesiology and Reanimation, Karaman Training and Research Hospital, Karaman, Turkey

**Keywords:** Laparoscopic cholecystectomy, Postoperative Pain, Nerve block, Oblique Subcostal Transversus Abdominis Plane Block, Ultrasonography, Modified thoracoabdominal nerve Block through the Perichondrial Approach

## Abstract

**Background:**

Laparoscopic cholecystectomy(LC) causes significant postoperative pain. Oblique subcostal transversus abdominis plane(OSTAP) block was described for postoperative analgesia, especially for upper abdominal surgeries. Modified thoracoabdominal nerves block through perichondrial approach(M-TAPA) block is a new technique defined by the modification of the thoracoabdominal nerves through perichondrial approach (TAPA) block, in which local anesthetics are delivered only to the underside of the perichondral surface. The primary aim of this study was to evaluate the effect of M-TAPA and OSTAP blocks as part of multimodal analgesia on postoperative opioid consumption in patients undergoing LC.

**Method:**

The present study was designed as a randomized, controlled, prospective study. Seventy-six adult patients undergoing LC were randomly assigned to receive either bilaterally M-TAPA or OSTAP block after the induction of anesthesia and before surgery using bupivacaine 0.25%, 25 ml. The primary outcome was assessed as postoperative 24 h opioid consumption, between groups were compared. Secondary outcomes were Numerical Rational scale(NRS) scores, time to first opioid analgesia, patient recovery, using the Quality of Recovery-15 (QoR-15) scale, nausea and vomiting, sedation score, metoclopramide consumption, and evaluating the analgesic range of dermatome.

**Results:**

The mean tramadol consumption at the postoperative 24th hour was higher in the group OSTAP than in group M-TAPA (P = 0.047). NRS movement score at 12th hour was statistically significantly lower in group M-TAPA than in group OSTAP (P = 0.044). Dermatomes showed intense sensory analgesia between T7-11 in both groups, and it was determined that there was proportionally more involvement in the group M-TAPA. There were no differences between the groups in terms of other results.

**Conclusions:**

After the LC surgery, ultrasound-guided M-TAPA block effectively reduced opioid consumption, postoperative pain, and QoR-15 scores similar to OSTAP block.

**Clinical trial registration:**

The study was registered prospectively at clinicaltrials.gov (trial ID: NCT05108129 on 4/11/2021).

**Supplementary Information:**

The online version contains supplementary material available at 10.1186/s12871-023-02106-z.

## Background

Laparoscopic Cholecystectomy (LC) is a frequently performed surgery as the gold standard in the treatment of symptomatic gallstone disease [1]. Although LC is considered to be minimally invasive, it can cause moderate-severe pain in the postoperative period [2].

Poorly controlled early postoperative pain impairs the quality of recovery, increasing the risk of postoperative pulmonary complications as a risk factor for the formation of chronic pain [3]. It was observed that the total abdominal pain following LC mostly originates from the incision area, and the remaining part consists of visceral and referred pain [2, 4]. Multimodal analgesia, including opioids, is used to limit pain following LC [4]. Treatment with opioids might cause side effects e.g., Postoperative Nausea and Vomiting (PONV), respiratory depression, and constipation [5]. Neuraxial analgesia is rarely used in LC surgeries because of possible complications and technical difficulties [6]. Ultrasonography (USG)-guided Erector Spinae Plan (ESP) block and Oblique Subcostal Transversus Abdominis Plane (OSTAP) block are among the interfacial plane blocks used in LC. It was observed that the OSTAP block forms a block between T7-L1 in the anterior abdominal wall [7–9].

The perichondrial approach for blockage of Thoracoabdominal Nerves (TAPA) block is a novel block applied to the lower and upper surfaces of the chondrium by administering local anesthesia [10]. Modified Thoracoabdominal Nerves Block Through Perichondrial Approach (M-TAPA) block is another new technique defined as a modification of TAPA block in which local anesthetics are administered only to the underside of the perichondrial surface. It was previously reported that this technique creates a sensory block between T5-T12 dermatomes [11]. It has recently been used for postoperative analgesia in laparoscopic abdominal surgeries because it is considered to provide effective analgesia in the anterior and lateral thoracoabdominal walls [11, 12].

The primary purpose of the present study was to investigate the effect of M-TAPA and OSTAP blocks applied prior to surgery as part of multimodal analgesia on postoperative opioid consumption in patients scheduled to undergo LC. The secondary aims of the study were to evaluate the incidence of complications, postoperative pain degrees, first rescue analgesic administration time, dermatome areas where sensory analgesia was provided, and Quality of Recovery-15 (QoR-15) scores at the postoperative 24th hour between the groups.

## Methods

### Study design

**Fig. 1:**
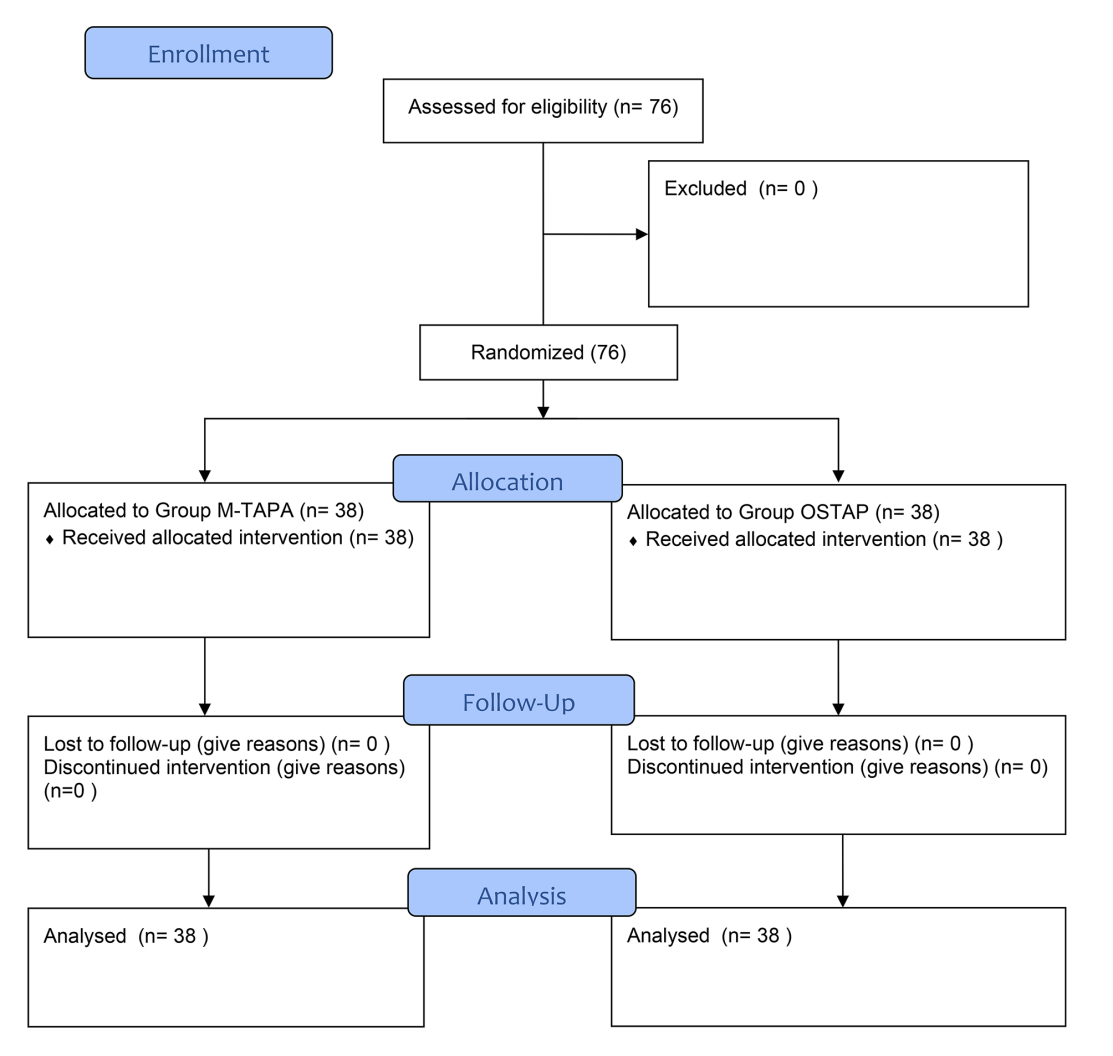
CONSORT diagram of study

The present study was planned in the prospective, double-blind, randomized controlled study design, and Institutional Review Board approval (07-2021/02) of Karamanoglu Mehmetbey University Faculty of Medicine, Turkey was obtained on October 11, 2021. The study was registered prospectively at clinicaltrials.gov (NCT0510812) on November 4, 2021 and designed in accordance with the principles set out in the Declaration of Helsinki. Written informed consent was obtained from all participants regarding the interventions and enrollment in the study. The Consolidated Standards for Reporting Studies (CONSORT) checklist was used for patient enrollment (Fig. 1). In this respect, patients, who were aged 18–70 years, assessed with American Society Anesthesiologists Physical Status (ASA) I-II, and scheduled for elective LC were included in the study. Presence of coagulation disorder, block injection site infection, known allergy to local anesthetics, advanced liver or kidney failure, previous history of abdominal surgery or trauma, conversion of laparoscopic surgery to open surgery, preoperative use of any pain reliever within 24 h, chronic opioid use, alcohol use or drug use, refusal to participate, inability to communicate in Turkish, pregnancy and Body Mass Index(BMI) ≥ 35 kg m^− 2^ were the exclusion criteria.

### Anesthesia application

Standard monitoring was performed with the measurement of non-invasive blood pressure, electrocardiography, and peripheral oxygen saturation in all patients in the operating room. Following the insertion of a 22-gauge intravenous (iv) line by the operating room anesthesiologists, iv fentanyl (1–2 µg kg^− 1^) and 1% propofol were administered by titration until unconsciousness. Endotracheal intubation was performed after muscle relaxation with iv rocuronium bromide 0.6-1 mg kg^− 1^. Mechanical ventilation was achieved using a pressure-controlled mode to maintain end-tidal carbon dioxide at 35 to 40 mmHg. Anesthesia was maintained by the infusion of sevoflurane (0.8-1 minimum alveolar concentration) and iv remifentanil in an O_2_/air mixture (FiO_2_ 0.40). Remifentanil infusion was adjusted as 0.01–0.2 µg kg^− 1^ min^− 1^ to keep the mean arterial blood pressure of the patients within 20% of the baseline.

The standard 4-trocar method was used in the surgeries (superior umbilicus, epigastric region under the xiphoid, and right midclavicular, right anterior axillary line within sub-costal area) that were performed by the same surgeon on all patients, and the gas pressure was kept at 12 mm Hg to create the pneumoperitoneum. Before the remifentanil infusion was discontinued, 1 mg kg^− 1^ iv tramadol was administered to all patients at the end of surgery. Following the completion of the surgery, the patients were extubated and transferred to the post-anesthesia care unit (PACU). The patients were discharged to the ward when the Modified Aldreth Score ≥ 8.

### Patient randomization

The patients, who were included in the study, were divided randomly into Group M-TAPA and Group OSTAP according to the computerized randomization table created by a researcher who was not involved in the study. A random code was assigned to each patient, who participated in the study, to ensure blindness, and each code was placed in sealed envelopes. The surgery room anesthetist took from a file the corresponding sealed envelope indicating the block to be performed for each randomized patient.

Block applications were performed in both groups following routine anesthesia induction and patient intubation. (Supplementary Fig. 1) In this way, the patients were kept blind to the groups. The block injection sites were closed with the same dressing in both groups.

Surgeons and surgery room anesthetists were not included in any postoperative stage, including data collection, data access, and data analysis. The researchers who were interested in recording postoperative parameters were masked in the distribution of patient groups and did not have access to randomization until data analysis was complete.

### Block interventions

Following the tracheal intubation, for Group M-TAPA, a high-frequency linear probe (6–13 MHz) was placed in the sagittal direction on the 10th costal margin under the guidance of USG to define the transversus abdominis, internal oblique, and external oblique muscles. The probe was placed sagitally on the 10th costal margin in the midline and a deep angle was given to the costochondral angle. The lower costal cartilage was visualized in this way. By using the in-plane technique, a 22G, 80 mm block needle (Stimuplex B-Braun Medical, Melsungen, Germany) was placed in the cranial direction between the transversus abdominis muscle and the lower surface of the costal cartilage by moving the needle tip towards the posterior aspect of the 10th costal cartilage. Then, 25 ml of 0.25% bupivacaine was injected into the lower surface of the chondrium, making sure that the needle tip did not cross the cranial edge of the 10th costal cartilage. The same process was then repeated for the other side.

For Group OSTAP, the OSTAP block was applied to the patients in the supine position immediately following endotracheal intubation. The anesthesiologist of the surgery room placed the ultrasound linear probe along the subcostal margin of the upper abdominal wall and obliquely from the xiphoid to the right iliac crest in the midabdominal line. The rectus abdominis muscle and the underlying transversus abdominis muscle were identified near the costal margin. The needle was guided into the transversus abdominis fascia. 25 ml of 0.25% local anesthetic solution was injected between the rectus abdominis and transversus abdominis muscles along the oblique subcostal line. The same process was then repeated for the other side.

### Evaluation of pain

Numeric Rating Scale (NRS), which is a numerical scale in which the respondent selects an integer (0–10 integer) that best reflects the intensity of the pain, was used to assess the postoperative pain. In this regard, “0” meant “no pain” and “10” meant “worst pain imaginable”.

The NRS scores at rest and in motion were recorded by an anesthetist who did not know the group distributions at postoperative min 0, min 15, min 30, min 60, 2nd hour, 6th hour, 12th hour, and 24th hour. Patients were educated and familiarized with NRS scores at the preoperative period.

#### Postoperative analgesia

Postoperative analgesia was administered with iv paracetamol 1 g every 6 h and iv dexketoprofen 50 mg every 12 h to all patients. The first doses of drugs were administered following the completion of the block procedures, before the surgical incision. If the NRS score of the patient was ≥ 4, iv tramadol 50 mg was administered as a rescue analgesic. If the PONV score of the patient was ≥ 2, iv metoclopramide 10 mg was administered.

### Outcome measures and data collection

The primary purpose of the study was to compare the total opioid (tramadol) consumption at the postoperative 24th hour in the groups. Also, tramadol consumptions were recorded separately between 0 and 1 and between 1-12th, 12-24th hours.

In addition to these, the NRS score, time of first rescue analgesic administration, and QoR-15 questionnaire scores filled by the patient before and following the surgery were noted. Sedation, PONV Scores at the specified hours, antiemetic consumption, and thoracoabdominal areas with sensory block 2 h following surgery were also recorded.

The QoR-15 Scale is a Turkish-validated questionnaire that has been used to evaluate the quality of postoperative recovery and the health of patients in the early postoperative stages. Each item rates the responses to 15 subjective parameters on a scale of 0–10 with a minimum score of 0 and a maximum score of 150 [13]. A score of 0 represents the lowest improvement, and a score of 150 represents the excellent quality of recovery in the QoR-15 Questionnaire. Higher scores indicate a higher quality of recovery experience. All patients were asked to complete the QoR-15 Questionnaire twice, on the morning of the surgery, in the preoperative waiting area, and 24 h following the surgery.

PONV score was evaluated verbally with a descriptive scale (0 = None, 1 = Mild Nausea, 2 = Moderate Nausea, 3 = One Vomiting, 4 = Multiple Vomiting). The assessment of the sedation level was scored on a 4-point score (0 = wake, 1 = sleepy, easy to verbally awaken, 2 = sleepy, 3 = not opening eyes to verbal commands). PONV and sedation scores were recorded at the same time points as the NRS assessment.

A researcher who was not assigned to the surgery room evaluated the T3-L1 sensory levels 2 h following the surgery by using the Pinprick Test. Anterior and lateral cutaneous branches were evaluated on a vertical line 3–5 cm from the midline and midaxillary lines, respectively. (Supplementary Fig. 2) A 3-point numerical scale (0 = no pain, 1 = decreasing pain, 2 = normal pain) was used. The values ​​of 0 or 1 were defined as effective [14]. The normal sense in the shoulder was used for comparison. The first oral intake and mobilization times of the patients following surgery were also recorded.

### Statistical analysis

The data was statistically analyzed using the SPSS software (Version 22, SPSS Inc., Chicago, IL, USA). Numbers (n) and percentages (%) were used to represent categorical variables. Depending on the data normal distribution, descriptive statistics of numerical data were presented using the mean standard deviation or median quartiles (Q1, Q3). The Shapiro-Wilk test was used to determine whether the data had a normal distribution. Student’s t-test was used to compare normally distributed numerical data across two independent groups and the Mann-Whitney U test was used to compare non-normally distributed data. The Chi-square test or Fisher exact test was used to compare proportions between categorical variables. *P* < 0.05 was chosen as the statistical significance level.

### Sample size

The sample size was calculated according to the pilot study including 10 patients in each group. Postoperative %20 reduction of tramadol consumption at the 24th hour was accepted clinically meaningful. The mean tramadol consumption was 120 ± 34 in the group OSTAP and 95 ± 47 in the group M-TAPA. Therefore, assuming Type I error = 0.05 and Type II error = 0.2 (%80 power to detect this difference), then 32 patients were needed per each group. Considering dropouts, 38 patients were decided to include in each group.

## Results

A total of 76 patient data were analyzed in the present study. A total of 69.7% (n = 53) of the patients were female and 30.3% (n = 23) were male. The mean age of the patients was found to be 50.19 ± 10.61 (19–69). The comparison of the demographic and clinical characteristics of the study groups is given in Table 1. The demographic and clinical characteristics (i.e., gender, weight, height, body mass index, age, ASA, first oral intake, first mobilization, first opioid time, first metoclopramide time, anesthesia time, and surgery time) between the study groups were statistically similar (P > 0.05, Table 1).

**Table 1:** Comparison of demographic and clinical characteristics between research groups

		Group M-TAPA (n = 38)	Group OSTAP (n = 38)	*P* values
**Gender**	Male	12 (31.6%)	11 (28.9%)	0.803^a^
	Female	26 (68.4%)	27 (71.1%)	
**Age (years)**		47.94 ± 10.63	52.44 ± 10.23	0.06^c^
**Weight (kg)**		77.5 (72.25-85)	78 (71.5–85)	0.996^b^
**Height (cm)**		165 (162-173.5)	164 (160–170)	0.182^b^
**BMI (kg/m** ^**2**^ **)**		27.64 ± 2.65	28.16 ± 2.94	0.430^c^
**ASA**	1	15 (39.5%)	9 (23.7%)	0.139^a^
	2	23 (60.5%)	29 (76.3%)	
**First oral intake time**		7 (5-7.25)	7 (5–8)	0.792^b^
**First mobilization time**		5 (3–6)	5 (3–6)	0.971^b^
**First opioid time**		0.50 (0.25–7.25)	0.50 (0.25–1.25)	0.123^b^
**First metoclopramide time**		0 (0-6.25)	0.125 (0–2)	0.761^b^
**Duration of anaesthesia**		60 (50-66.25)	67.5 (55-76.25)	0.061^b^
**Surgical time**		50 (42.25-60)	60 (50-71.25)	0.103^b^

^a^Chi-Square test with n (%)

^b^Mann-Whitney U test with median (quartiles, Q1 - Q3)

^c^Student’s t test with mean ± standard deviation

(OSTAP: Oblique subcostal transversus abdominis plane; M-TAPA: Modified thoracoabdominal nerves block through perichondrial approach; ASA: American Society Anesthesiologists Physical Status; BMI: Body Mass Index)

The comparison of the postoperative rescue analgesic requirement rates and amounts (in mg) between the groups is given in Table 2. The mean tramadol consumption was higher in group OSTAP than group M-TAPA at the postoperative 24th hour (P = 0.047). The amount of postoperative rescue analgesic requirement was not statistically different between the groups at other time intervals (P > 0.05, Table 2).

**Table 2:** Comparison of postoperative rescue analgesic requirement rates and amounts (mg) between groups

	Frequency comparison			Milligram comparison		
Time frame (hour)	Group M-TAPA (n,%)	Group OSTAP (n,%)	*P* values	Group M-TAPA (mg)	Group OSTAP (mg)	*P* values
**0–1**	15 (39.5%)	22 (57.9%)	0.108^a^	0 (0–50)	50 (0–50)	0.119^b^
**1–12**	14 (36.8%)	19 (50%)	0.247^a^	0 (0–50)	25 (0–50)	0.179^b^
**13–24 (second 12 h)**	20 (52.6%)	23 (60.5%)	0.488^a^	50 (0–50)	50 (0–50)	0.359^b^
**0–24**	34 (89.5%)	32 (84.2%)	0.497^a^	50 (50–100)	100 (50–150)	**0.047** ^**b**^

^a^Chi-Square test with n (%)

^b^Mann-Whitney U test with median (quartiles, Q1 - Q3)

(OSTAP: Oblique subcostal transversus abdominis plane; M-TAPA: Modified thoracoabdominal nerves block through perichondrial approach)

**Fig. 2:**
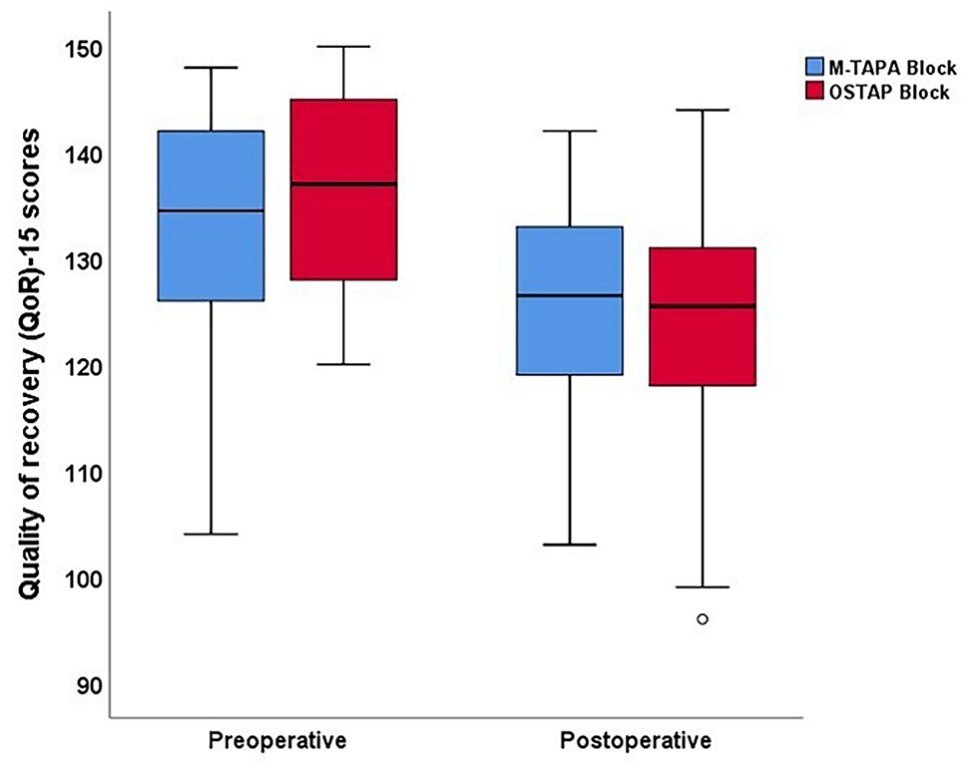
Box-plot of the distribution of the total scores of preoperative and postoperative quality of recovery (QoR)-15 between the groups. (OSTAP: Oblique subcostal transversus abdominis plane, M-TAPA: Modified thoracoabdominal nerves block through perichondrial approach.)

Preoperative and postoperative Quality of Recovery (QoR)-15 total scores were not statistically different between the study groups (P > 0.05). The box-plot of the distribution of preoperative (group M-TAPA, 132.94 ± 10.7; group OSTAP, 136.42 ± 8.6; p = 0.123) and postoperative (group M-TAPA, 125.63 ± 10.07; group OSTAP, 123.39 ± 11.61; p = 0.373) (QoR)-15 scores between the groups is given in Fig. 2.

**Fig. 3:**
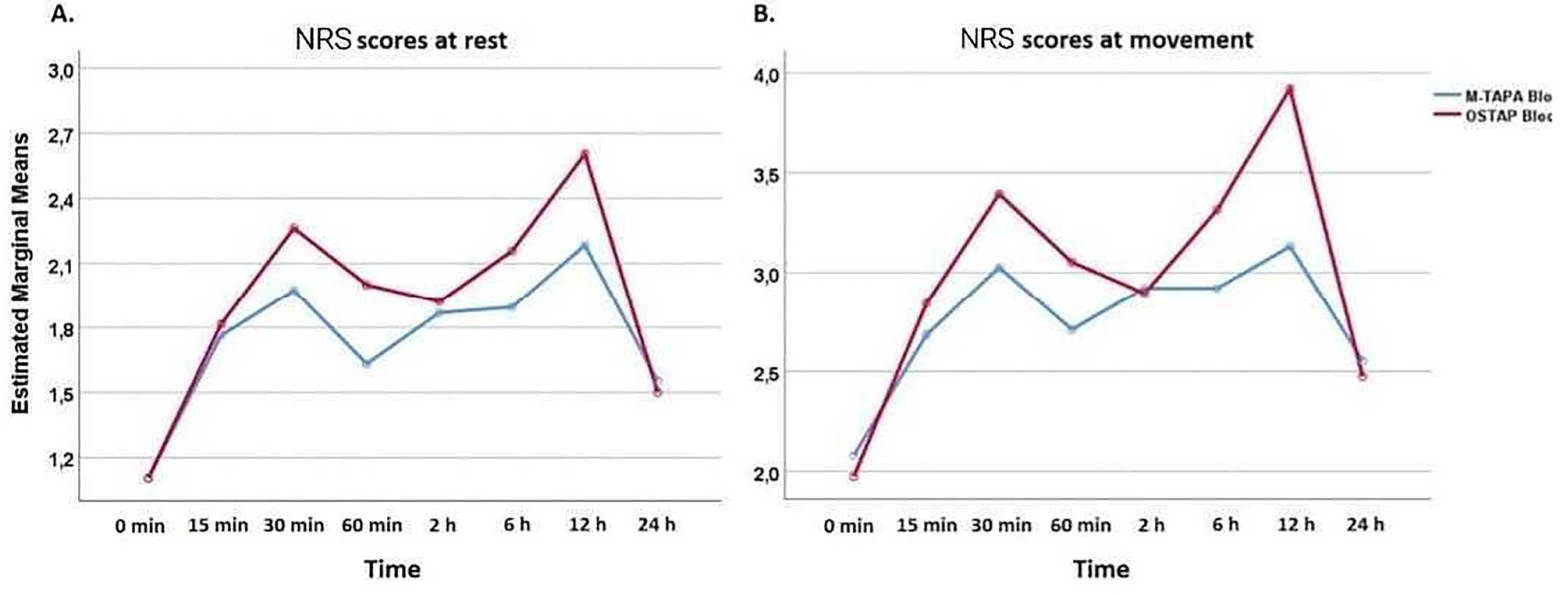
**A.** Time-dependent line graph of mean NRS (resting) scores for M-TAPA block and OSTAP block groups. **B.** Time-dependent line graph of mean NRS (movement) scores for M-TAPA block and OSTAP block groups. (OSTAP: Oblique subcostal transversus abdominis plane, M-TAPA: Modified thoracoabdominal nerves block through perichondrial approach, NRS: Numerical Rating Scale)

NRS movement score was statistically and significantly lower at the 12th hour in the group M-TAPA (P = 0.044). No statistically significant differences were detected between the groups in NRS scores measured in motion at other time intervals (P > 0.05). No statistically significant differences were detected in resting NRS scores between the groups at all time ranges (P > 0.05). A time-dependent line graph of the mean NRS (rest) and (movement) scores for group M-TAPA and group OSTAP is given in Fig. 3.

Figure 3 NRS change between the 15th minute and 24 h at rest and movement.

Sedation, PONV scores, and metoclopramide requirement amounts were not statistically different between the groups (P > 0.05).

**Fig. 4:**
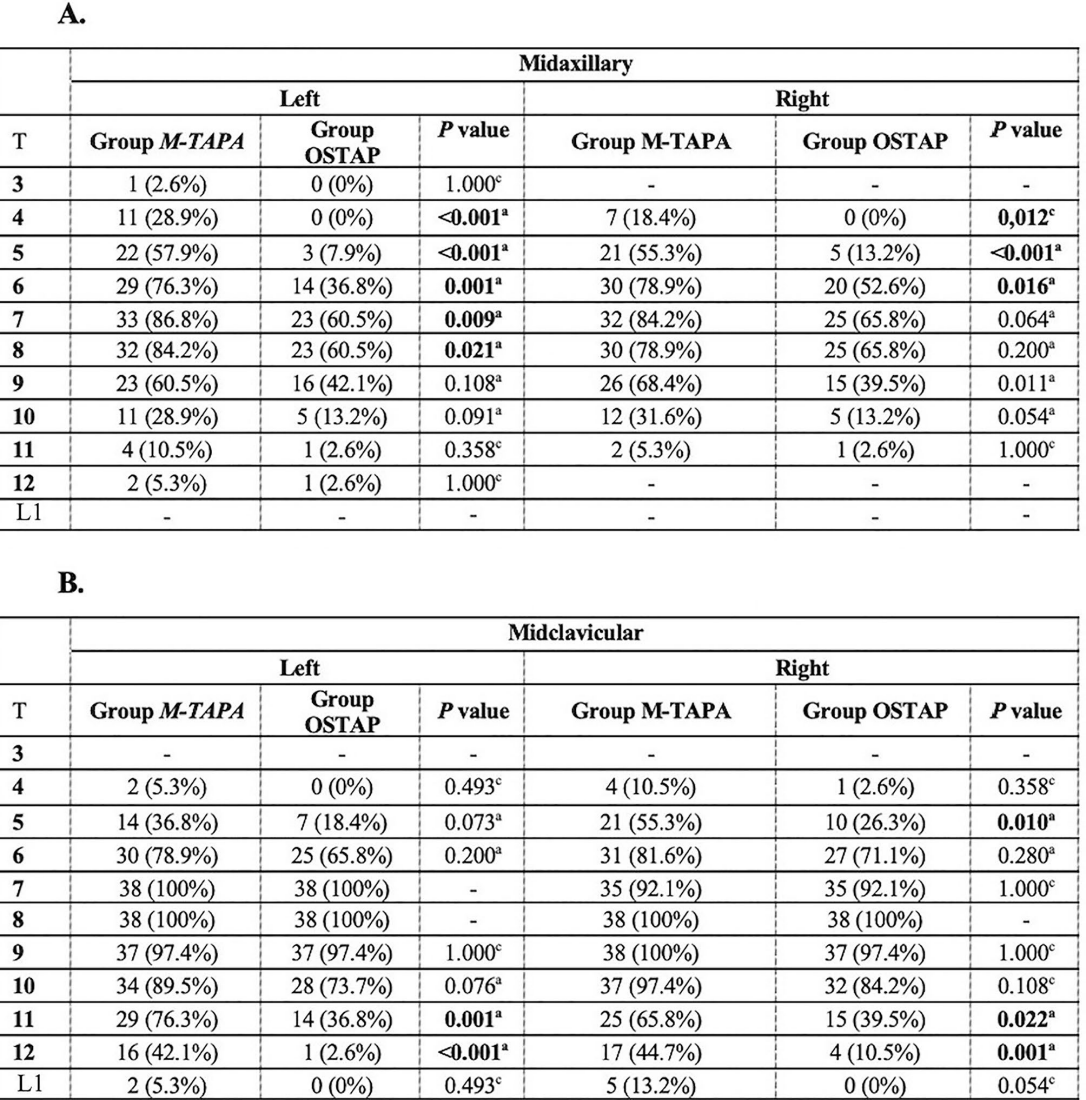
**A:** Number (percentage) of patients for each blocked dermatome in the Midaxillary line. **B.** Number (percentage) of patients for each blocked dermatome in the Midclavicular line. ^a^Chi-Square test with n (%) ^c^Fisher exact test with n (%) (OSTAP: Oblique subcostal transversus abdominis plane, M-TAPA: Modified thoracoabdominal nerves block through perichondrial approach)

The involvement rates for the left and right sides of the anterior and lateral regions between the M-TAPA and OSTAP groups are given in Fig. 4.

## Discussion

The present study is the first randomized, double-blind clinical trial that compared M-TAPA block with OSTAP block in LC. The study showed that USG-guided bilateral M-TAPA block following the induction of general anesthesia in LC patients reduced the total tramadol requirement in the first 24 h compared to the group OSTAP. Also, NRS scores were less than 4 at almost all time points and were similar in the groups. No differences were detected between the groups in terms of postoperative complications and quality of recovery. The number of dermatomes blocked in the anterior and lateral abdominal wall in group M-TAPA was higher than in group OSTAP.

Severe pain is associated with delayed postoperative ambulation, decreased patient satisfaction, development of chronic pain, and increased pulmonary and cardiac complications. For this reason, it has great importance to define optimal analgesic strategies for surgical procedures causing moderate-severe pain. It is important to understand the trigger and source of postoperative pain in LC. Most of the postoperative pain components (50–70%) originate from the incision sites following LC, and pneumoperitoneum (20–30%) and cholecystectomy (10–20%) also cause pain [4]. Bisgaard et al. reported that somatic pain associated with incision was more dominant than visceral pain [15]. Since postoperative pain is triggered by many reasons, pain control is provided with multimodal analgesia. It is argued that the use of regional anesthesia and analgesia in multimodal analgesia is important in pain conditions and to overcome the neuroendocrine stress response to traumas [16].

The anterior abdominal wall is primarily formed by the rectus abdominis muscle innervated by the T6-L1 spinal nerves [17]. Hebbard et al. defined OSTAP block, which is a variation of Transversus Abdominis Plane (TAP) block that provides supraumbilical analgesia under USG [7, 18]. OSTAP block is used as an effective regional anesthesia technique in middle and upper abdominal surgeries [18, 19]. It has a lower complication rate because of direct USG. Many previous studies showed that OSTAP block reduces postoperative pain and opioid consumption in LC [9, 20, 21]. It was also reported that OSTAP block improves respiratory function following LC [20]. Although the OSTAP block provided NRS scores below 5 at all time points in the present study, the M-TAPA block reduced postoperative tramadol consumption more than the OSTAP block.

It was reported that OSTAP block affects anterior abdominal wall predominantly with a limited extent lateral abdominal wall [8]. Despite the reported successful results in previous studies, the fact that the patchy sensory block pattern on the lateral and posterior abdominal walls in the patients treated with OSTAP block for LC caused discomfort and might have resulted in the consumption of high-dose tramadol. In their study, Lee et al. showed that OSTAP block affects four segments of the anterior abdominal wall with the cephalic T8 by using a volume of 20 mL [19]. In a previous study that was conducted on 12 healthy volunteers, the sensory block of the lateral and anterior areas of the abdominal wall was evaluated starting from the T6 dermatome level, and it was found that 26% of the lateral area and 90% of the mid-abdomen area were blocked in the OSTAP after administering 20 ml 0.375% ropivacaine [8]. In this study, the dermatome area was evaluated with the cold test, and the volunteers were not subjected to any abdominal surgery [8]. In our study, 25 ml 0.25% bupivacaine was administered before the incision for LC, and dermatome evaluation was done by pinprick test and we did not calculate the affected area. There was no involvement of lateral dermatomes in 11 (28.9%) patients in the Group OSTAP in our study. We explain this difference in the affected dermatomes as an application of relatively higher volume, surgical manipulations, or the effect of CO_2_ insufflation into the abdomen.

TAPA block, described by Tulgar et al., is a novel regional technique that provides analgesic effects in the anterior and lateral regions of the abdominal wall by injecting a local anesthetic into the lower and upper parts of the chondrium at the costochondral corner [10]. Modification of this block was named an M-TAPA block in which local anesthetic is applied to the lower border of the chondrium [11].

Various case reports showed that M-TAPA block leads to successful and effective postoperative analgesia in abdominal surgeries. It was reported that M-TAPA block leads to satisfactory analgesia without a prescription of opioid in a case operated with a midline incision below and above the umbilicus. Moreover, M-TAPA block and catheter placement was performed in 2 patients who underwent major abdominal surgery, and it was found to be beneficial [11, 22]. In different series in which the M-TAPA block was applied, the blockade of dermatomes was observed between T7-T11, T5-T10, and T3-T12 [11, 12, 23]. In these cases, it was also observed that the postoperative analgesic efficacy could be prolonged up to 24 or even more hours, and opioid consumption decreased. It is considered that the prolongation of the analgesia for up to 36 h in interfacial plane blocks is because of the decreased absorption of local anesthetic in an environment with low vascularity [24]. This might be a possible explanation for the prolonged analgesia seen in our patients in both groups. In this respect, M-TAPA might be an option for analgesic management in patients recruited for abdominal surgery with an extended analgesia time.

Various predictions for the mechanism of action of M-TAPA block were made previously in cadaveric studies. In their cadaver study, Tanaka et al. mentioned the existence of a “tunnel” structure between the costal cartilage and the origin of the Transversus Abdominis Muscle (TAM). Since this part was found stained with dye after dissection at the end of the study, they explained the spread of local anesthetic agents to the cranial side without obstruction of the linea semilunaris [14]. It was found that the stain did not spread on T7, but they argued that there might be more cranial spread than T7 because of various factors such as pneumoperitoneum in humans, the intraoperative position being upside down in laparoscopy, and the intra-abdominal pressure. Again, in the study that was conducted by Ciftci et al., it was reported that the stain spread between T4-11/12 [25]. Aikawa et al. detected the highest sensory level in the median (IQR) of Dermatomes as T7 (T5–8) in the anterior and T9 (T7-10) in the lateral region in 30 patients who underwent gynecological surgeries, sensory loss was observed in the lateral region in five patients [26]. Also, Zinboonyahgoon et al. reported that a single LA injection under the ribs could achieve multilevel intercostal nerve block, which might play role in the responsible mechanism of M-TAPA [27].

In the present study, it was found that M-TAPA block could spread from T4 to L1 in the anterior region, T3-T12 Dermatomes were blocked in the lateral region in some patients, and no loss of sensation was observed in the lateral region in 4 patients.

The deeper fibers of the anterior and lateral cutaneous branches of the 7–11 thoracic intercostal nerves, which partially innervate the parietal peritoneum might affect visceral pain [28]. The visceral pain’s severity and duration depend on the surgical procedure’s duration, insufflation pressure and duration, and complete or partial evacuation of the pneumoperitoneum [28]. OSTAP and M-TAPA blocks lead to blockage of dermatomes between T7-11. It was considered that it might contribute less to the palliation of visceral pain in addition to parietal pain. Effective pain relief was achieved in both blocks in the first 24 h of the postoperative period. In both groups, NRS values ​​were lower than 4 at all times. However, postoperative 24-hour tramadol consumption was less with M-TAPA block in this study. We think that more dermatome involvement and sensory block in a wider area would be responsible for the reduction of opioid consumption.

In anesthetized patients, postoperative recovery is a multidimensional and reticular process with several interrelated areas and is not limited to postoperative pain. For this reason, the QoR-15 questionnaire, which is a Turkish-validated post-surgical quality assessment tool that can compare patient-centered outcomes to assess and improve the quality of care, was created [13, 29]. Although we did not evaluate the real effect of both blocks on QoR15 scores due to lack of control group in the study design, it could be mentioned that there was no difference in postoperative recovery scores between the groups. Pain-assisted complications that might occur after LC can be prevented after M-TAPA and OSTAP blocks. In the present study, puncture site infection, abdominal organ injury, and local anesthetic toxicity did not occur, showing that these blocks are safe when performed under USG guidance. On the other hand, the aforementioned interfascial block-related complications should be kept in mind during perioperative period [30].

The study was conducted to show the postoperative pain and quality of recovery between a novel technique, M-TAPA block, and OSTAP block, and to compare the consumption of postoperative analgesic agents. The results showed no difference except for 24-hour opioid consumption and showed that M-TAPA and OSTAP blocks had similar effects. This report is relatively recent and the study is the first controlled study comparing OSTAP block with a new and different regional anesthesia technique, M-TAPA block.

The study had several limitations. Although morphine consumption is generally used as a comparison in studies evaluating regional anesthesia techniques, tramadol was preferred in the study since it was shown that it has the same effects as morphine in patients undergoing LC, the effects are seen in a shorter time and the side effect profile is less [31, 32]. Another limitation was that our study did not record intraoperative analgesia consumption and depth of anesthesia and incidence of shoulder pain at the postoperative period. On the other hand, remifentanil is a rapidly metabolized short-acting opioid, so the residual effect of it can be ignored during the postoperative period. Moreover, the addition of adjuvants like alpha-2 agonists, dexamethasone, and ketamine to local anesthetic to increase the efficiency of the blocks would be a further research topic of the M- TAPA block. Also, the performance time and easiness of the blocks were not evaluated.

The sample size of the present study was calculated for the minimum number of patients required for tramadol consumption to find a statistically significant difference in the results. However, there was insufficient power to analyze common adverse events related to nausea and vomiting, such as PONV and sedation scores with the current sample size.

## Conclusion

It was found in the present study that adding M-TAPA block, which is a novel intraoperative technique for multimodal analgesia, reduces opioid consumption because of extensive dermatome involvement up to 24 h following LC surgery.

## Electronic supplementary material

Below is the link to the electronic supplementary material.

Supplementary Material 1

Supplementary Material 2

## Data Availability

The datasets used and/or analyzed during the current study are available from the corresponding author on reasonable request.

## Abbreviations

M-TAPA: Modified thoracoabdominal nerve block through the perichondrial approach
LC: Laparoscopic cholecystectomy
OSTAP: Oblique subcostal transversus abdominis plane block
TAPA: Thoracoabdominal nerves through perichondrial approach
USG: Ultrasonography
ESP: Erector spinae plane
PONV: Postoperative nausea and vomiting
QoR-15: Quality of Recovery-15
ASA: American Society Anesthesiologists
LA: Local anesthetic
CONSORT: Consolidated Standards of Reporting Trials
BMI: Body mass index
IV: Intravenous
PACU: Post-anesthesia care unit
NRS: Numerical rating scale
TAP: Transversus abdominis plane
TAM: Transversus abdominis muscle
