# Supplementary material for: Comparing ultrasound-guided modified thoracoabdominal nerves block through perichondrial approach with oblique subcostal transversus abdominis plane block for patients undergoing laparoscopic cholecystectomy: a randomized, controlled trial
Source: BMC Anesthesiol. 2023 Apr 27;23:139. doi: 10.1186/s12871-023-02106-z (PMC10134575; doi:10.1186/s12871-023-02106-z)
Supplement: Supplementary file 1 — Supplementary Material 1 [file 12871_2023_2106_MOESM1_ESM.docx]

Supplementary figure 1


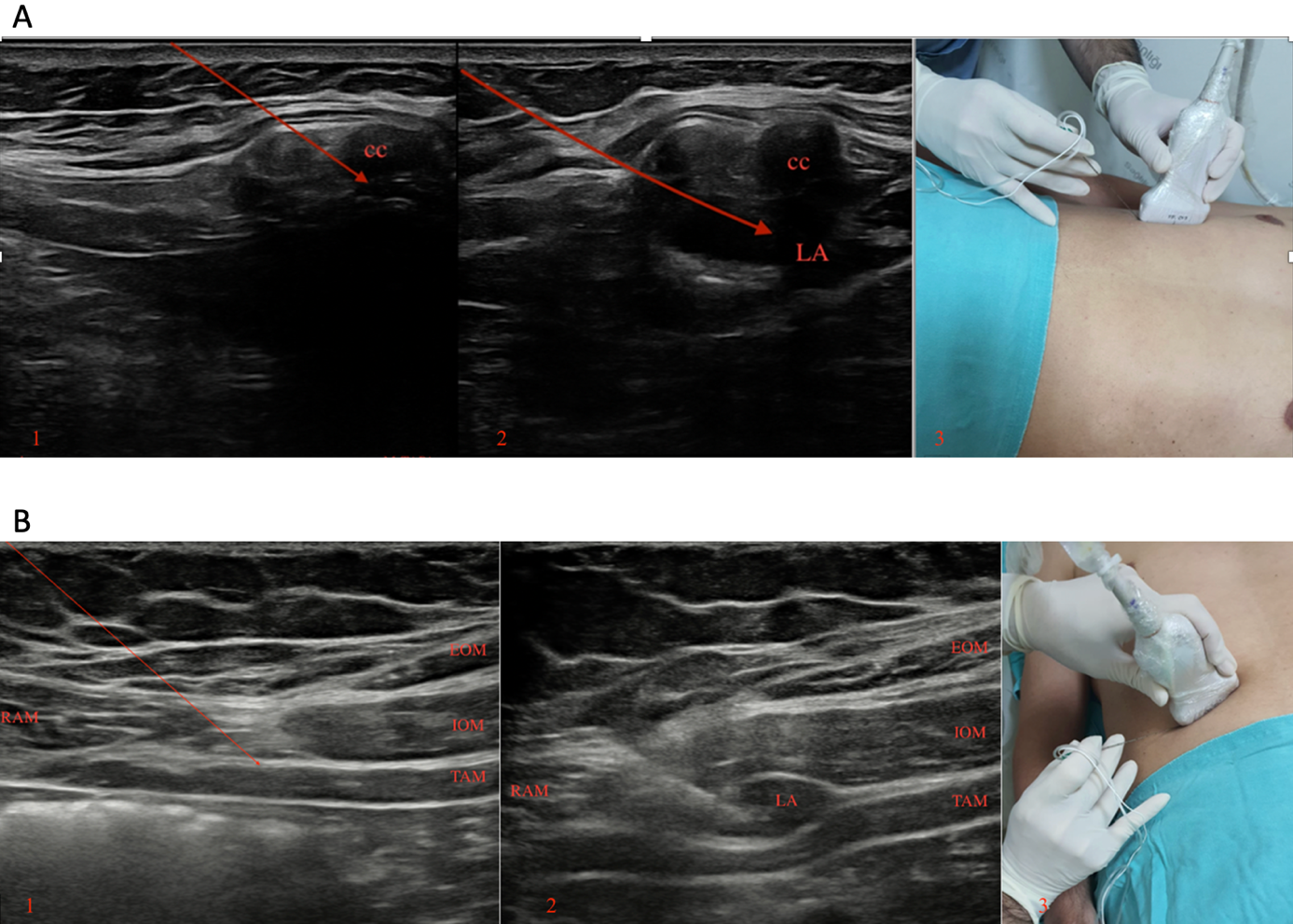


Image of the probe position and an ultrasound image of the blocks

A: Modified-thoracoabdominal nerve block through a perichondrial approach Block

(1) Ultrasound image of the perichondral area before blocking

(2) Sonographic view of local anesthetic injection point at the lower aspect of the chondrium.

(3) Image of the probe position

(cc: costal cartilage, LA: Local anesthetic).

B: Oblique subcostal transversus abdominis plane block

(1) Ultrasonographic view of the abdominal muscles

(2) Sonographic view of the block needle and injection point at the oblique subcostal transversus abdominis plane block

(3) Image of the probe position

(EOM: external oblique muscle, IOM: internal oblique muscle, TAM: transversus abdominis muscle, RAM: Rectus abdominis muscle, LA: local anesthetic)
