# Supplementary material for: Comparing ultrasound-guided modified thoracoabdominal nerves block through perichondrial approach with oblique subcostal transversus abdominis plane block for patients undergoing laparoscopic cholecystectomy: a randomized, controlled trial
Source: BMC Anesthesiol. 2023 Apr 27;23:139. doi: 10.1186/s12871-023-02106-z (PMC10134575; doi:10.1186/s12871-023-02106-z)
Supplement: Supplementary file 2 — Supplementary Material 2 [file 12871_2023_2106_MOESM2_ESM.docx]

Supplementary figure 2


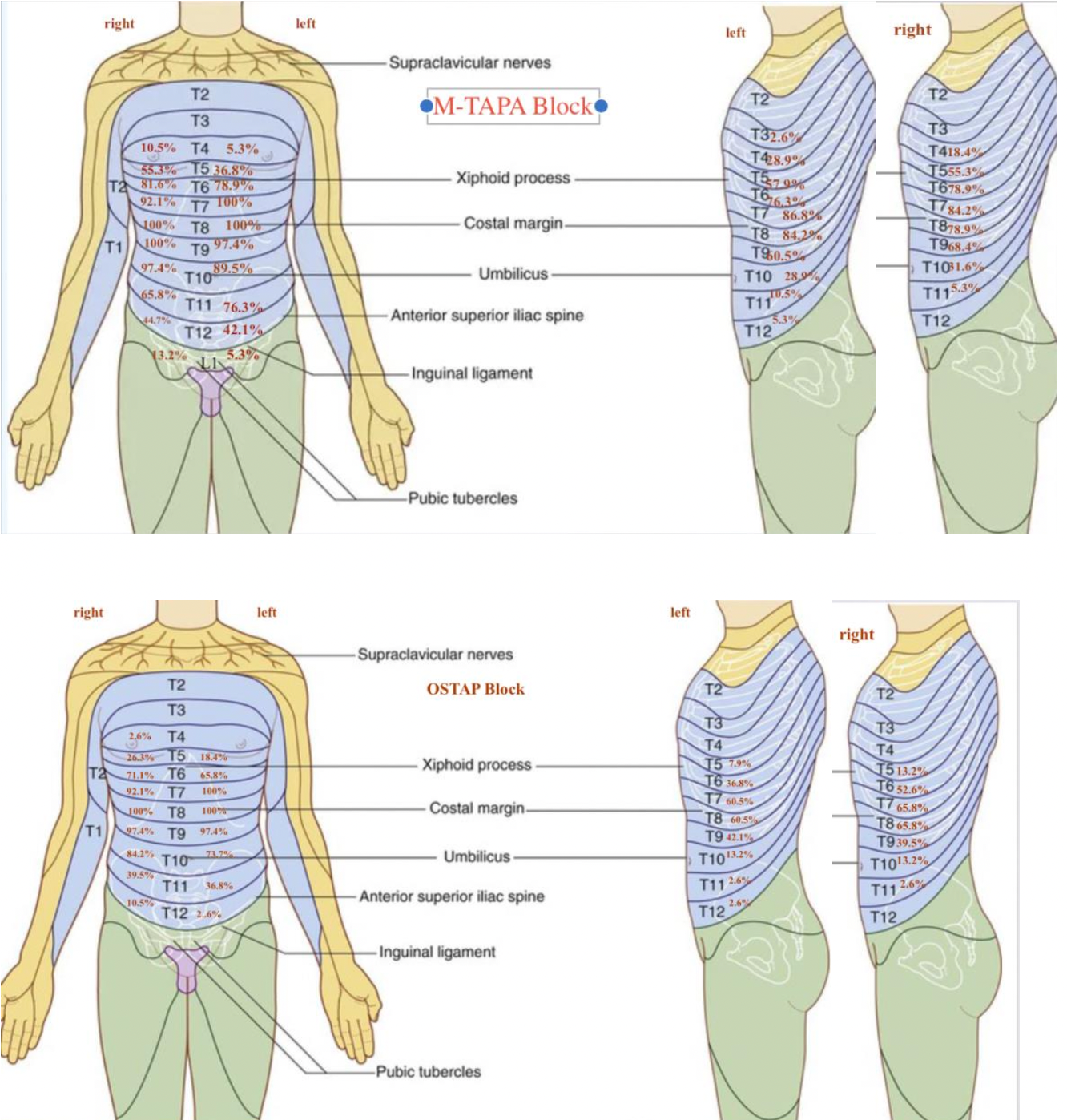


The figures showed the percentage of patients for each blocked dermatome in the midaxillary and midclavicular lines. (M-TAPA Block: Modified-thoracoabdominal nerve block through a perichondrial approach Block, OSTAP Block: Oblique subcostal transversus abdominis plane block )
